# Autoassociative Memory and Pattern Recognition in Micromechanical Oscillator Network

**DOI:** 10.1038/s41598-017-00442-y

**Published:** 2017-03-24

**Authors:** Ankit Kumar, Pritiraj Mohanty

**Affiliations:** 1 Department of Physics, California Institute of Technology, 1200 E. California Blvd, Pasadena, CA 91125 USA; 2 Department of Physics, Boston University, 590 Commonwealth Avenue, Boston, MA 02215 USA

## Abstract

Towards practical realization of brain-inspired computing in a scalable physical system, we investigate a network of coupled micromechanical oscillators. We numerically simulate this array of all-to-all coupled nonlinear oscillators in the presence of stochasticity and demonstrate its ability to synchronize and store information in the relative phase differences at synchronization. Sensitivity of behavior to coupling strength, frequency distribution, nonlinearity strength, and noise amplitude is investigated. Our results demonstrate that neurocomputing in a physically realistic network of micromechanical oscillators with silicon-based fabrication process can be robust against noise sources and fabrication process variations. This opens up tantalizing prospects for hardware realization of a low-power brain-inspired computing architecture that captures complexity on a scalable manufacturing platform.

## Introduction

Inspired by studies that have indicated that subsystems of the brain involved in associative learning exhibit synchronization dynamics by which pattern recognition emerges from the frequency entrainment of the constituent oscillating neurons^1^, significant recent interest has developed around the prospect of constructing analogous systems using artificial, physical oscillators. Such systems of coupled physical oscillators are capable of autoassociative memory operation and other forms of parallel, non-Boolean and neuromorphic computing, and suitably engineered, offer the advantages of far higher operating frequencies than their biological counterparts, and far lower power requirements than attempts to simulate neural networks on traditional hardware.

The dynamics of a system of coupled oscillators can exhibit attractive limit cycles that represent synchronized states. Information can be stored in either the phase or frequency differences between oscillators at synchronization, and retrieved through dynamical flow to these attractors. Following the initial proposals by Hoppensteadt and Izhikevich^2–4^, recent work has focused on schemes to implement the basic principle across a variety of different platforms. Spin-torque oscillators, in which the magnetization of a thin ferromagnetic layer is induced into sustained oscillation through the application of bias current or external magnetic field, have been shown to be capable of frequency locking via a number of methods, and show promise as a platform for neurocomputing^5–9^. Similarly, vanadium oxide relaxation oscillators, which rely on a precise switching between metallic and insulating states^10^, have been successfully synchronized using capacitive coupling with aims towards application to associative memory operation^11^. Other studies have focused on RRAM^12^ and anchored disk resonators^13^.

Here, we consider MEMS (Micro-Electro-Mechanical Systems) resonators^14^ configured to self-oscillate as the oscillating elements our artificial neural network. MEMS resonators have found significant use as sensors, biomedical implants, and wireless communication devices due to their high operating frequencies and low power requirements. They have already been explored as a platform for reversible computation^15^ and probing the thermodynamic limits of computation^16^, and logic circuits^17, 18^. Self-oscillation of a MEMS resonator^19^ with unprecedented phase noise and thermal stability and phase synchronization between two such self-oscillators^20, 21^ have been successfully demonstrated. As the required fabrication methods are based on standard lithography and processing techniques currently in use at semiconductor foundries, any architecture based on an array of MEMS oscillators would easily lend itself to highly scalable manufacturing. Furthermore, MHz to GHz range operation frequency will enable computing or pattern recognition at very high speed. For these reasons, MEMS devices have always been considered a desirable architecture for analog computing^22^.

In this article, we present detailed simulation results based on a realistic physical model that captures the dynamics of these resonators. In contrast to previous work^23^ on a network of physical oscillators, our work is the first to consider the ensemble effects of nonlinearity, frequency and coupling strength dispersion, and noise. Our study focuses on constraining the ranges of these effects over which synchronization and pattern retrieval remain robust. In particular, we find that stringent control over the frequency dispersion across the oscillator array will be crucial in practical realization.

## Model

Each individual MEMS self-oscillator is modeled as a Van der Pol Duffing oscillator:

1 $$\ddot{x}+{\lambda }\dot{x}({x}^{2}-1)+{\omega }_{0}^{2}x(1+\kappa {x}^{2})=0$$

Here, ω_0_ is the natural oscillation frequency of the self-oscillation system. The damping function *x* ^*2*^ − *1* enables self-sustained oscillation. The parameter λ controls the amplitude of self-oscillation, whereas κ controls the nonlinear, anisochronous behavior.

**Figure 1:**
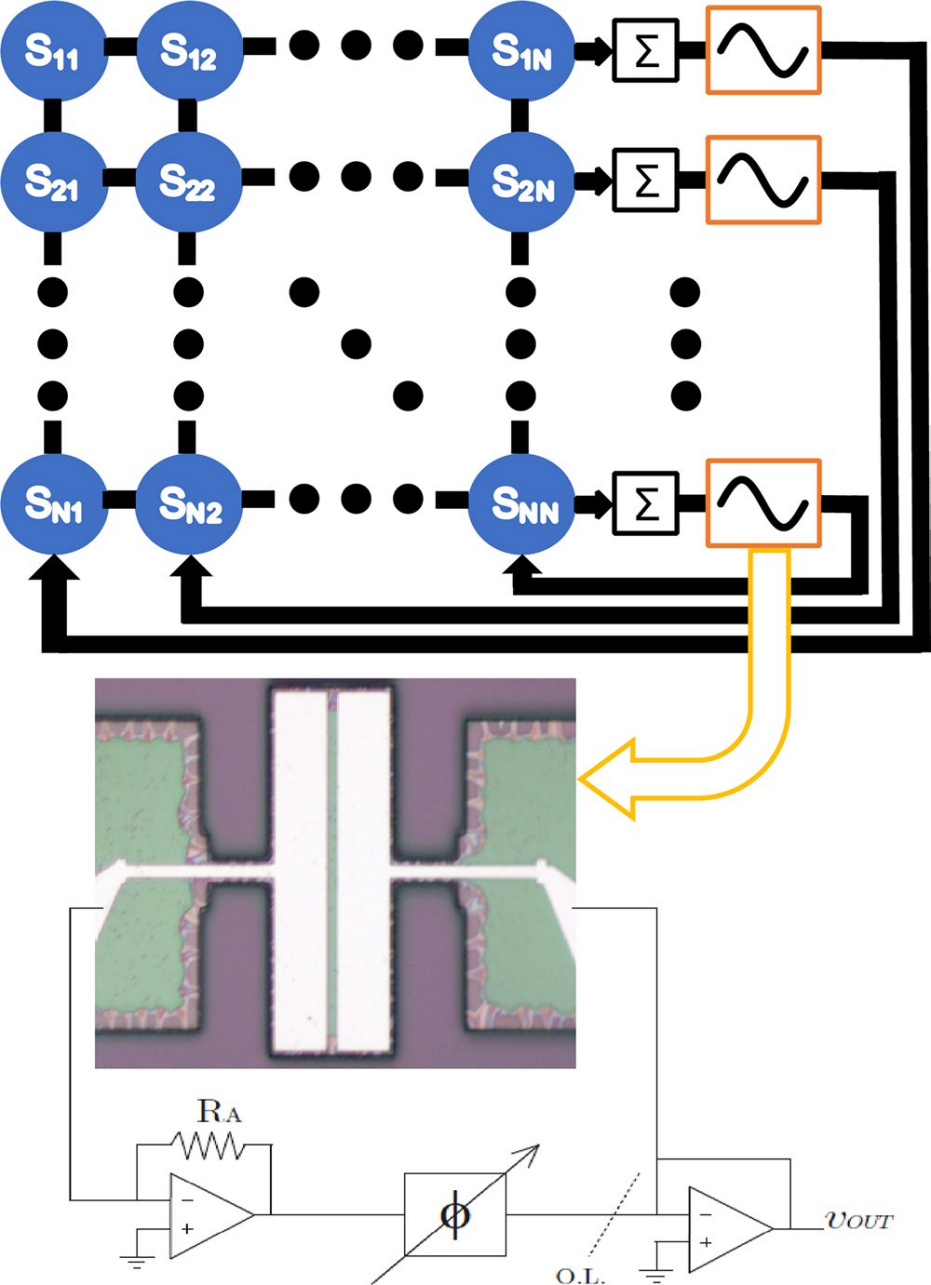
Schematic of oscillator network with all-to-all coupling. Signals across the array are summed with weights determined by equation (8). Each individual self-oscillator is comprised of a MEMS resonator (optical micrograph of a plate-type resonator shown below) placed within a feedback loop with a transimpedence amplifier, phase shifter, and output buffer that satisfies Barkhausen’s criterion.

A system of *n* oscillators is then connected by linear all-to-all coupling, as shown in the schematics in Fig. 1, where we allow for both dissipative (velocity) and reactive (displacement) coupling. This gives the equations of motion for the *i* ^*th*^ oscillator as:

2 $${\ddot{x}}_{i}+\lambda {\dot{x}}_{i}({x}_{i}^{2}-1)+{\omega }_{i}^{2}{x}_{i}(1+\kappa {x}_{i}^{2})=\sum _{j=1}^{n}({p}_{ij}{\dot{x}}_{j}+{q}_{ij}{x}_{j})$$

We take coupling to be symmetric: ${p}_{ij},{q}_{ij}={p}_{ji},{q}_{ji}$. The individual natural oscillation frequencies are allowed to vary. Displacements are rescaled according to $\tilde{x}=\sqrt{\lambda x}$ and time according to $\tilde{t}=\omega t$. Given this change of variables, the equations of motion now read:

3 $${\ddot{\tilde{x}}}_{i}+{\dot{\tilde{x}}}_{i}({\tilde{x}}_{i}^{2}-\lambda )+{{\rm{\Omega }}}_{i}^{2}{\tilde{x}}_{i}(1+\tilde{\kappa }{{\tilde{x}}_{i}}^{2})=\sum _{j=1}^{n}({\tilde{p}}_{ij}{\dot{\tilde{x}}}_{j}+{\tilde{q}}_{ij}{\tilde{x}}_{j})$$

where $\tilde{\kappa }$ and the coupling constants have been rescaled accordingly. The frequencies Ω_*i*_ have been normalized to unity. In what follows we consider both identical oscillator frequencies and frequency dispersion, where each Ω_*i*_ is randomly chosen on the interval [1 − δ, 1 + δ]. We drop the tildes and persist with the dimensionless form from here on out.

In the absence of coupling terms, the dynamical system described by equation (3) exhibits stable limit cycle behavior via an Androponov-Hopf bifurcation for *λ* > 0. Accordingly, for small λ, the system can be transformed to a topologically equivalent normal form. The derivation is given in the Supplementary Information. This results in an equation for the complex amplitude *a*(*t*):

4 $${\dot{a}}_{i}=(\frac{\lambda }{2}-i\frac{{\omega }^{2}-{\omega }_{i}^{2}}{2}){a}_{i}-(\frac{1}{8}-\frac{3i\kappa {\omega }_{i}^{2}}{8\omega }){a}_{i}{|{a}_{i}|}^{2}+\sum _{j=1}^{n}(\frac{{p}_{ij}}{2}+\frac{{q}_{ij}}{2i\omega }){a}_{j}$$

By introducing the substitution ${a}_{i}={A}_{i}{e}^{i{\varphi }_{i}t}$, equation (4) can be written in amplitude-phase form. Furthermore, by measuring each *ϕ*_*i*_ relative to *ϕ*_1_ (the choice is arbitrary), we can reduce the dimensionality of the resulting system by one. Thus, let Δ*ϕ*_*i*_ = *ϕ*_*i*_ − *ϕ*_1_.

5 $${\dot{A}}_{i}=-\frac{1}{8}{A}_{i}^{3}+\frac{\lambda }{2}{A}_{i}+\sum _{j=1}^{n}\frac{{p}_{ij}}{2}\,\sin ({\rm{\Delta }}{\varphi }_{j}t)+\frac{{q}_{ij}}{2}\,\cos ({\rm{\Delta }}{\varphi }_{j}t)$$

6 $${\rm{\Delta }}{\dot{\varphi }}_{i}=\frac{{\omega }_{i}^{2}-{\omega }_{1}^{2}}{2\omega }+\frac{3\kappa {\omega }_{i}^{2}}{8\omega }{A}_{i}^{2}-\sum _{j=1}^{n}(\frac{{p}_{ij}}{2}\frac{{A}_{j}}{{A}_{1,2}}+\,\cos ({\rm{\Delta }}{\varphi }_{j}t)+\frac{{A}_{2,1}}{{A}_{1,2}}\,\cos ({\rm{\Delta }}{\varphi }_{j}t))$$

These equations represent an amplitude-phase coupled model that goes beyond the phase-only Kuramoto model that is often considered in the literature^24^. Bifurcations in the two-oscillator case have been the subject of both analytic and numerical study^25, 26^. The two-oscillator case exhibits bistability in a subset of the synchronization tongue where the in-phase and 180° out-of-phase oscillatory modes are both accessible depending on the supplied initial conditions. This simple model therefore already exhibits the ability to store information in the phase differences at synchronization.

In earlier works, this system^27, 28^ was studied in the opposite limit of a very large number of oscillators. Here, distributions in oscillator frequencies even with relatively narrow width can inhibit synchronization. These systems then demonstrate transitions towards global synchronization as the coupling strength between oscillators is varied that are not unlike second order phase transitions commonly studied in statistical physics.

The global stability of limit cycles in equation (4) can be proven in the absence of natural oscillation frequency mismatches and nonlinearity.

For κ = 0, and all *ω*_*i*_ = *ω*, equation (4) reads:

7 $$\dot{{a}_{i}=\frac{\lambda }{2}{a}_{i}-\frac{1}{8}{a}_{i}{|{a}_{i}|}^{2}+\sum _{j=1}^{n}(\frac{{p}_{ij}}{2}+\frac{{q}_{ij}}{2i\omega }){a}_{j}}$$

The stability of the set of fixed points ${a}_{i}^{o}$ of the complex amplitudes implies stability of limit cycles for the original displacement *x*. This existence of such a set can be proven without explicitly solving for the dynamical trajectories through the construction of a Lyapunov function, which can be considered to be a generalized energy function for the system. Such a real valued function $U(\{{a}_{i}(t),{a}_{i}^{\ast }(t)\})$ must be positive definite and satisfy $\frac{d}{dt}U(\{{a}_{i}(t),{a}_{i}^{\ast }(t)\})\le 0$ for a neighborhood around the critical points (${a}_{i}^{\ast }$ denotes complex conjugation). The following function satisfies these criteria^3^:

8 $$U=-\sum _{i=1}^{n}(\frac{\lambda }{2}{|{a}_{i}|}^{2}-\frac{1}{16}{|{a}_{i}|}^{4}+\sum _{j=1}^{n}(\frac{{p}_{ij}}{2}+\frac{{q}_{ij}}{2i\omega }){a}_{i}^{\ast }{a}_{j})$$

We note that U is bounded by $\frac{1}{16}{|{a}_{i}|}^{4}\,$ for large *a*_*i*_, and satisfies the relations ${\dot{a}}_{i}=-\frac{\partial U}{\partial {a}_{i}^{\ast }}$, ${a}_{i}^{\ast }=-\frac{\partial U}{\partial {a}_{i}}$. This allows us to write:

9 $$\frac{dU}{dt}=\sum _{i=1}^{n}(\frac{\partial U}{\partial {a}_{i}}{\dot{a}}_{i}+\,\frac{\partial U}{\partial {a}_{i}^{\ast }}{\dot{a}}_{i}^{\ast })=-2\sum _{i=1}^{n}{|{\dot{a}}_{i}|}^{2}\le 0$$

The quantity $\frac{dU}{dt}=\,0$ when all ${\dot{a}}_{i}=0$. Thus, the fixed points ${a}_{i}^{o}$ of ref. 4 are stable.

The requirement of *κ* = 0 can be relaxed if we are able to add to the damping function a term proportional to $\dot{x}{x}^{2}$ that cancels the cubic displacement term in the normal form transformation, but the authors are not aware of any practical means of implementing such functional forms of damping. For a mesoscopic number of oscillators with realistic dynamics such as those considered here, analytical methods are therefore largely intractable and simulation presents itself as the most useful tool for analysis.

We seek to store information in states of synchronized oscillation in which the relative phase differences across the array remain fixed. Let the patterns to be stored be given by the set of vectors ${\xi }_{i}^{\mu },\,\mu =1,\ldots ,m;\,\,i=1,\ldots ,n,\,{\xi }_{i}^{\mu }\in {\boldsymbol{C}}$ (see ref. 1). Then, assuming these patterns to be equilibrium solutions to equation (4), one can attain a formula for the coupling matrix^29^:

10 $$S=\rho P{P}^{ {\dagger } }$$

where *ρ* is an overall scaling on the coupling strength, ${P}_{ij}={\xi }_{i}^{j}$ and *P* ^†^ is the pseudo-inverse of *P*, $\frac{1}{n}{(\frac{1}{n}{\tilde{P}}^{T}P)}^{-1}{\tilde{P}}^{T}$, with $\tilde{P}$, *P* ^*T*^ denoting the complex conjugate and transpose of *P*, respectively. Note that in terms of the original coupling terms present in equation (3), $Re({S}_{ij})=\,{p}_{ij}/2,Im({S}_{ij})={q}_{ij}/2\omega$. In what follows, we will consider binary patterns, so that each ${\xi }_{i}^{\mu }={e}^{i\pi }\,{\rm{or}}\,{\xi }_{i}^{\mu }={e}^{2i\pi }$, but “grayscale” patterns can conceivably be assigned as complex valued phasors and stored through a mixture of both dissipative and reactive coupling in the physical system.

We define the current state of the system to be ${X}_{i},i=1,\ldots ,n$ such that if the phase of the *i* ^*th*^ oscillator is given as ${\varphi }_{i}=\arctan (\dot{x}/x),{\varphi }_{i}\in \,[0,\,2\pi ]$, then *X*_*i*_ = cos *ϕ*_*i*_. The overlap, *M*, with the stored pattern *μ*, is the projection of the current state onto *ξ* ^*μ*^:

11 $${M}^{\mu }=\frac{1}{N}|\sum _{i=1}^{n}{\xi }_{i}^{\mu }{X}_{i}|$$

In storing information in the stable fixed points of an energy function, our system exhibits the essential features of a Hopfield network^30^. The upper bound storage capacity of Hopfield networks is known to scale linearly with n, the number of constituent network elements^31^. This upper bound is saturated by a completely orthogonal pattern set $(\sum _{i=1}^{n}{\xi }_{i}^{\mu }{\xi }_{i}^{\eta }=0,\,\forall \mu ,\eta )$. In the case of n orthogonal patterns, the expression for the coupling coefficients given by equation (10) reduces to an uncoupled network *S*_*ij*_ = *δ*_*ij*_. In practice, attempting to store even fewer patterns than this may yield a coupling matrix that gives rise to subnetworks (i.e. the network fractures into subnetworks that are decoupled from each other). Such network segmentation in the presence of incommensurate frequencies can greatly hinder phase synchronization, since phase locking requires frequency entrainment, yet such entrainment in general is impossible without at least indirect coupling between every oscillator. This somewhat limited storage capacity is a fundamental limitation of our reliance on dynamical fixed points.

## Absence of Noise

### Identical Oscillator Frequencies

We consider a system of 96 oscillators, arranged into an 8 × 12 grid for the purpose of visualization. The 26 patterns spanning the English alphabet shown in Figure S1 are stored, with black elements corresponding to ${\xi }_{i}^{\mu }=-1$, and white elements to ${\xi }_{i}^{\mu }=1$. The overlap between the stored patterns, $M=\,\frac{1}{N}|\sum _{i=1}^{n}{\xi }_{i}^{\mu }{\xi }_{i}^{\eta }|,\,\,$ ranges between 0 (orthogonal) to 0.854.

The degree of overlap with the set of stored patterns is tracked during the simulation. While evolution towards a phase-locked, synchronized state is desired due to the long timescale stability it provides, pattern recognition is achieved if, after a finite simulation time, the degree of overlap is greater than that of any other pattern. In what follow, phase differences are measured relative to *ϕ*_1_. Except where noted, the initial conditions of the array are set as the “a” pattern subject to random pixel flips. Simulation and analysis is conducted using MATLAB software.

**Figure 2:**
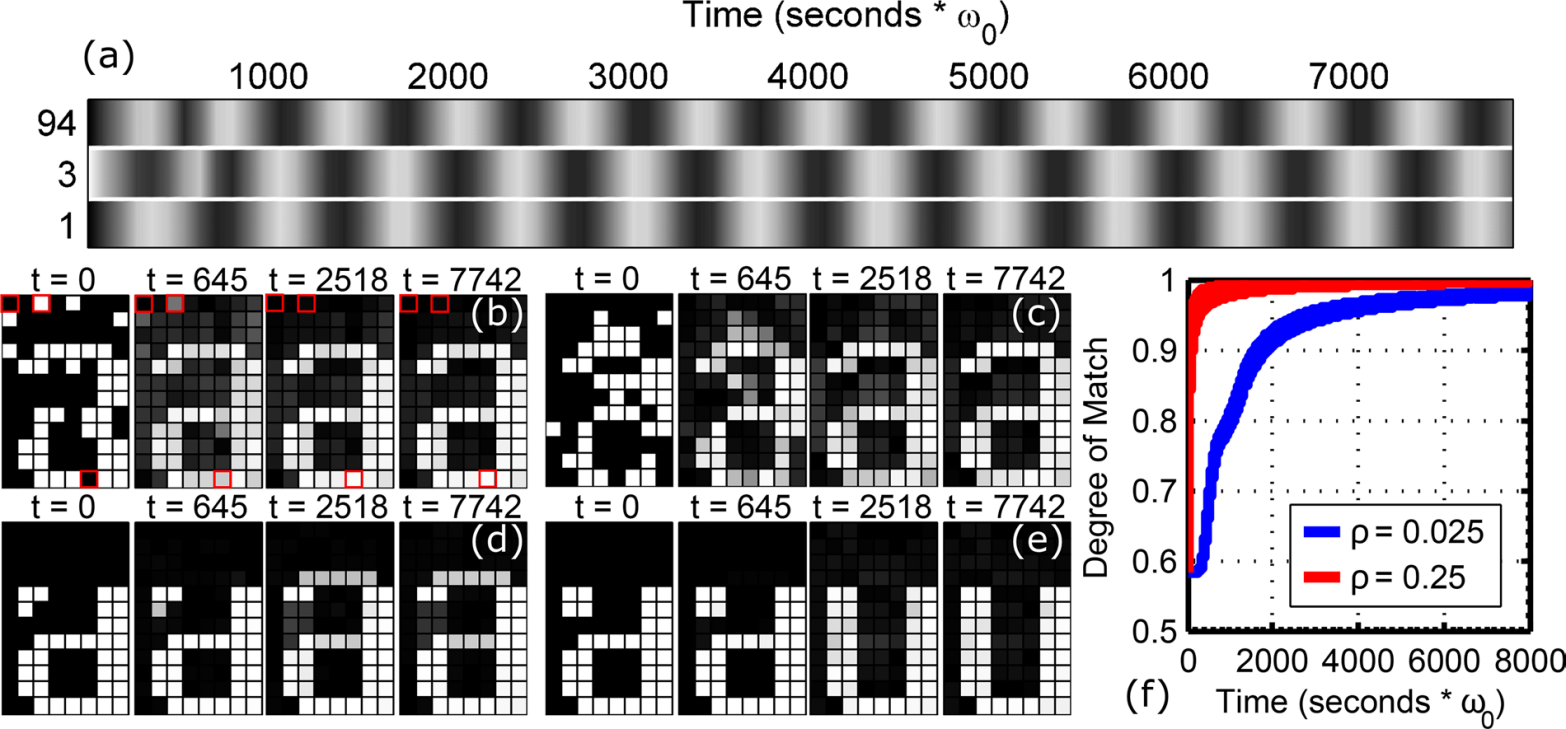
In the absence of distributed frequencies and nonlinearity, the system evolves predictably from the initial conditions, always selecting the stored pattern with the highest degree of match with the initial conditions. In figures (**b**) and (**c**), recognition of the “a” pattern is achieved even with significant distortion. Figure (**a**) shows the entire time series of the three highlighted pixels in (**b**). Pixels 1 and 97 start in phase but end out of phase. Pixels 1 and 3 start out of phase but end in phase. Figures (**d**) and (**e**) demonstrate the borderline case where recognition transitions from the stored “a” pattern to the stored “u” pattern. For similar patterns, significant distortion is not required before this transition occurs. Figure (**f**) demonstrates the effect of increasing the coupling strength *ρ* by an order of magnitude. The time needed to achieve a high degree of match is dramatically reduced.

In the absence of nonlinearity, noise, and distributed frequencies, the array evolves from initial conditions in a predictable manner; i.e. the array always progresses to a store synchronized state that corresponds to the highest degree of match with the initial pattern, shown in Fig. 2. Once dynamical flow has converged to one of the stable limit cycles, stability is guaranteed by the existence of a Lyapunov function (equation (8)). Synchronization is rapid, occurring within a few hundred cycles. As shown in Fig. 2e, the recognition time can be quickened further by increasing the overall coupling strength scale between oscillators.

We next increment the nonlinearity parameter *κ*. The effects are twofold: the degree of match parameter exhibits oscillations of a characteristic shape, and the response of the oscillator system stiffens in the sense that flow towards the synchronization state occurs even more rapidly. The amplitude of oscillations in the degree of match parameter generally grows with increasing *κ* and the effect is noticeably dependent on the overall coupling strength scaling. Simulations run for 80,000 oscillator cycles indicate long-term stability of synchronization, in the sense that the amplitudes of degree of match oscillations do not grow substantially (Figure S2).

### Frequency and Coupling Strength Dispersion

We next introduce distributed frequencies, randomly selecting from a uniform distribution over the interval [1 − δ, 1 + δ]. In the two-oscillator case, 1:1 frequency entrainment between two oscillators in the presence of detuning between their natural oscillation frequencies occurs within a region of parameter space known as the Arnold’s tongue^26, 32^.

**Figure 3:**
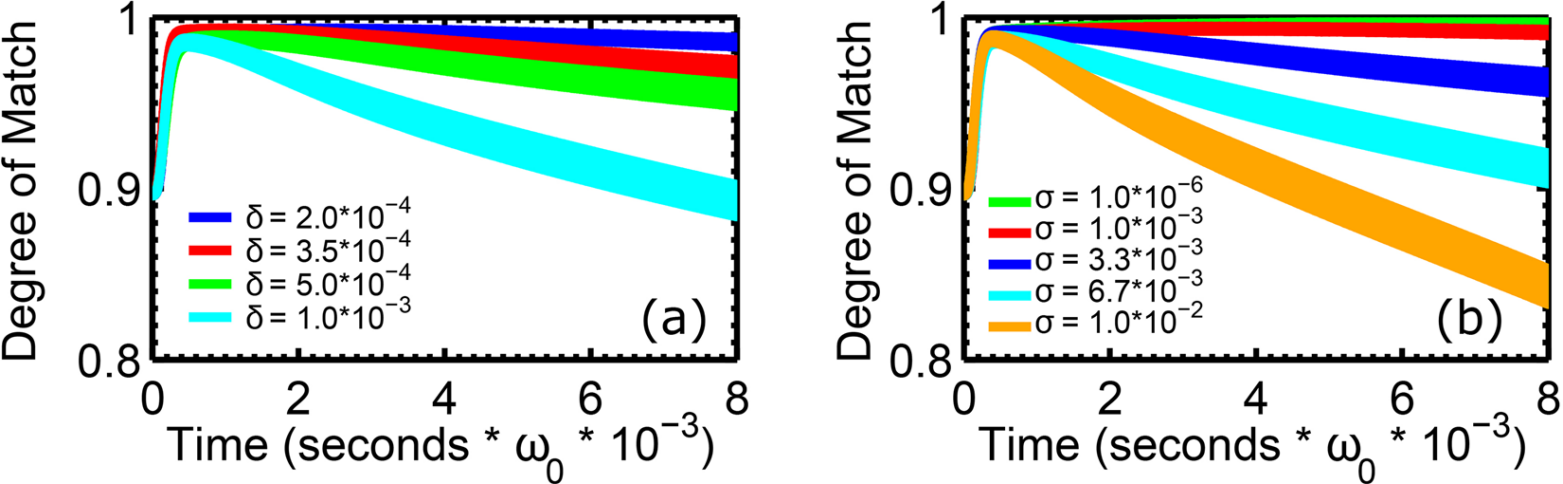
(**a**) Degree of match with the “a” pattern as the width of the uniform distribution from which oscillator frequencies are selected is widened. Stable phase locked synchronization does not occur, and the effect worsens with widening width (denoted in figure legend). (**b**) Plot of degree of match with the “a” pattern for uniform oscillator frequencies, but iterated *σ* (shown in the legend). Stability of the degree of match parameter is reasonable until *σ* = 3.3 × 10^−3^. This is an order of magnitude larger than the typical value of *δ* that destroys effective synchronization, suggesting that the system is more robust under coupling strength dispersion than frequency dispersion. In both (**a**) and (**b**), the shown curves have been averaged across 100 independent simulations, and then subsequently over 100 time steps; larger oscillations in degree of match parameter for wider dispersion widths are still visible.

In our 96-oscillator system, dynamical flow in the presence of distributed frequencies approaches the stable phase-locked states, but over time it loses its coherence. The effect worsens as the distribution is widened (Fig. 3a), with a significant dropoff in the degree of match for *δ* = 1.0 ∗ 10^−3^. Our simulations indicate that increasing the coupling strength does not ameliorate recognition, in contrast to result in the two oscillator case at fixed detuning, where the synchronization tongue widens with increasing coupling strength^26^. Note the thicknesses in the degree of match curves are fast timescale oscillations in the parameter.

Introducing nonzero *κ* again stiffens the response of the system, effectively quickening the rate of its dynamics. This is demonstrated in Figure S2, but it should be noted that the presence or absence of nonlinearity does not significantly affect the magnitude or stability of the degree of match parameter over time.

**Figure 4:**
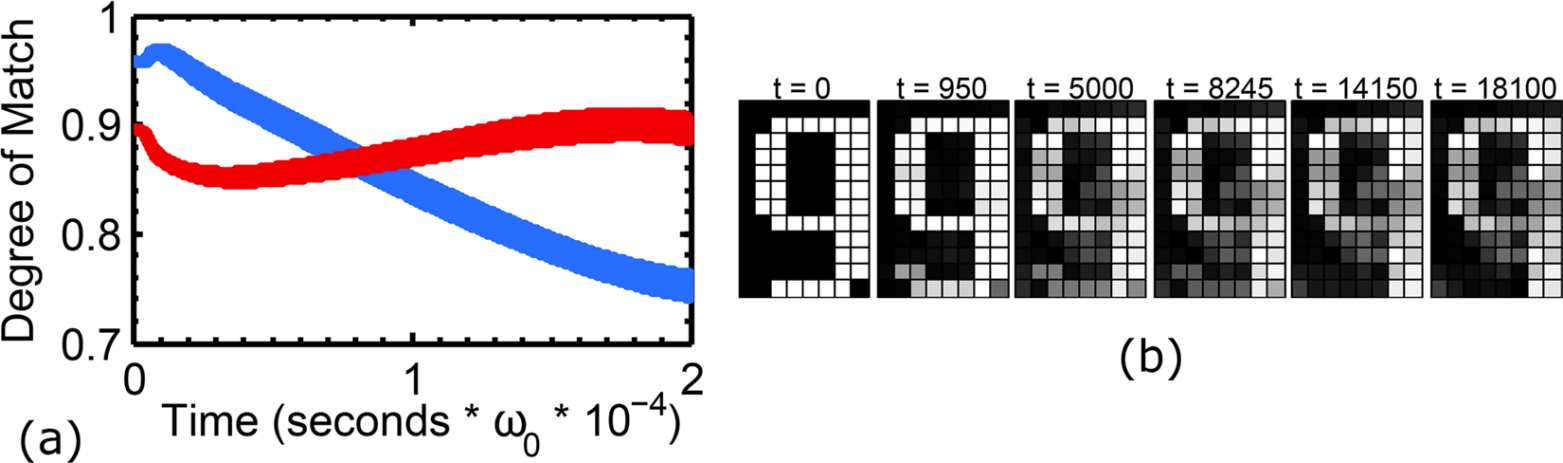
(**a**) Degree of match with the “g” pattern (blue) and “p” pattern (red). The initial conditions favor the “g” pattern, so the expected behavior is for the system to evolve towards a stable high degree of match, but the presence of frequency dispersion leads to a loss of stability, and eventually recognition of the “p” pattern occurs instead. Simulation used *δ* = 10^−3^ (**b**) Visualization of the same simulation. Second panel corresponds to the maximum of the blue curve in (**a**), last panel to the maximum of the red curve.

This loss of stability over long time periods poses a serious challenge to accurate pattern retrieval in the case when two or more patterns feature high overlap. Without distributed frequencies, initial conditions will evolve towards the configuration with the higher degree of match, but in its presence, the loss of stability over time can lead to selection of the other pattern (Fig. 4). If the distribution of frequencies is broad enough, then the stored patterns need not even be highly overlapping; the system will be unable to distinguish between a wide number of patterns; pattern recognition in the absence of phase synchronization with widely distributed frequencies is therefore unreliable.

Physically, frequency dispersion arises from variability in the resonator fabrication process. Coupling strength dispersion is motivated by similar considerations; through whatever means coupling is achieved (mechanical, capacitive, radiation), perfect precision is elusive in an experimental setting. Given this, as the encoding of patterns as stable fixed points in the dynamics is accomplished by a particular coupling matrix (equation (10)), determining whether pattern retrieval is reasonably robust in the presence of deviations from the learning rule is a relevant question for implementation that can only reasonably be answered with simulation.

To this end, we introduce defects into the coupling matrix by modifying each *p*_*ij*_ in equation (3) by uniform random variables chosen from a distribution centered at 0 with width *σ*. Sample results in Fig. 3 demonstrate that the effect is similar to, but less severe with regards to the magnitude of *δ* vs. *σ*, in the sense that large *σ* clearly prevents phase locking from occurring, and the degree of match parameter diminishes over time. A marked decline is present at *σ* = 1.0 ∗ 10^−2^. It should be pointed out, however, that regardless of the long-term performance, the degree of match invariably peaks for the correct pattern early on, suggesting that a scheme where recognition is defined on the basis of this earliest peak may be reliable.

## Addition of Noise

We are further able to demonstrate robustness in the presence of small degrees of noise independently perturbing each oscillator. The term ${\epsilon }{\eta }_{i}(t)$ is added to the right hand side of equation (3), where ${\epsilon }$ is a small parameter giving an overall scaling on the noise amplitude, and each *η*_*i*_(*t*) is a Gaussian white noise source with *η*_*i*_(*τ*_1_)*η*_*j*_(*τ*_2_) = *δ*_*ij*_ *δ*(*τ*_1_ − *τ*_2_). Simulation of the system with reasonable memory requirements is achieved through the implementation of a stochastic Runge-Kutta method^33^.

We first simulate the effects of white noise on a single self-sustaining oscillator. In the absence of such noise, such an oscillator will clearly exhibit a single peak in its frequency response. The addition of noise introduces additional power at the sidebands, and will suppress power at the main oscillation frequency (See Supplementary Information). The addition of sideband noise becomes salient at ${\epsilon }=0.01$ and significant suppression of the primary frequency is evident at noise amplitudes another order of magnitude larger. Analyzing the response in phase space defined by $(x,\dot{x})$, where *x* is the displacement of the oscillator, a noiseless self-sustaining oscillator will converge rapidly to a limit cycle. Introducing increasingly large amplitudes of noise will perturb the oscillator from this limit cycle until periodicity is almost entirely lost (see Supplementary Information).

**Figure 5:**
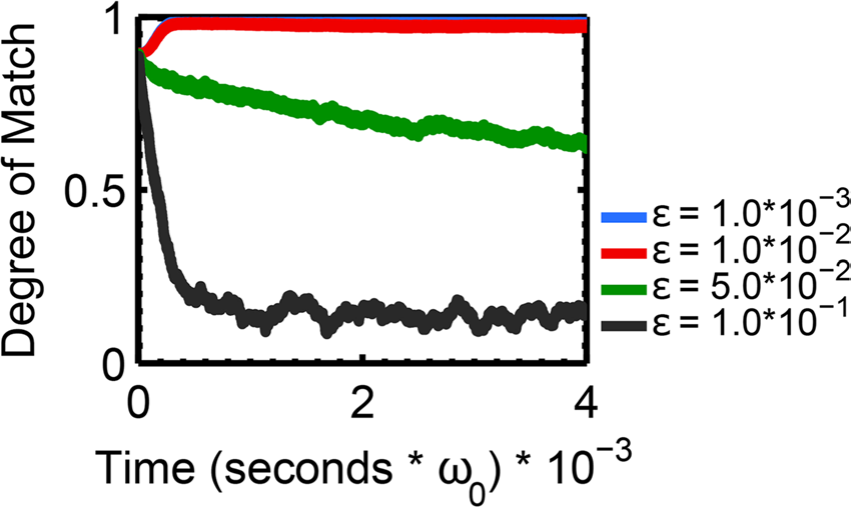
Plot of degree of match with the “a” pattern for varying levels of noise, averaged over ten realizations. Simulations contain *κ* = 0.1, *δ* = 10^−4^, *σ* = 10^−4^, *ρ* = 0.025. At noise levels up to ${\epsilon }={10}^{-2}$, the degree of match parameter remains stable over time. At higher noise levels, the system is unstable. These noise amplitudes correspond to the highly aperiodic regime (as shown in Supplementary Information), so the failure to synchronize is expected.

In a coupled array, the effects of noise are manifest in the dynamics of each oscillator and propagate across the array through the coupling. Still, we find synchronization to be fairly robust in the presence of white noise. For perturbation amplitudes on the order of 10^−2^, the degree of match parameter remains stable over time (Fig. 5). Noise amplitudes higher than this comprise several percent of the self-sustaining amplitude of the oscillator, which has been normalized to unity in this simulations by setting *λ* = 0.1. This regime, in which synchronization is rapidly lost, occurs where each oscillator itself deviates significantly from its limit cycle (Figure S3).

Taken together, the constraints on permissible *δ*, *σ* and ${\epsilon }$ before synchronization becomes unstable over long timescales yield order of magnitude estimates of the parameter ranges within which the basins of attraction of the fixed phase configurations remain attractive. A physical realization of such an oscillator array must be engineered to perform within these ranges.

## Micromechanical Self Oscillators

We now provide brief details regarding the physical realization of an array of MEMS self-oscillators, the guiding implications of the results of our numerical simulations.

At the core of each self-oscillator is the MEMS resonator itself. One specific example could be a piezoelectric resonator, fabricated by standard SOI silicon process, where a layer of a piezoelectric material such as aluminum nitride is grown on top of a suspended silicon layer. These types of devices exhibit low motional resistance at high frequencies. In particular, the primary longitudinal (length extension) mode has relatively low frequency, aiding electronic design, higher piezoelectric coupling, and higher vibrational amplitude, which would facilitate direct mechanical (reactive) coupling between resonators.

The resonance frequency of this mode is determined by the length of the resonator. When piezoelectrically actuated, this resonance frequency also corresponds to $1/\sqrt{{L}_{m}{C}_{m}}$, where *L*_*m*_ and *C*_*m*_ are the motional inductances and capacitances of the resonator, respectively.

The beam dynamics are described by the following Lagrangian^34^:

12 $$ {\mathcal L} =\frac{1}{2}m{L}^{2}{\dot{x}}^{2}(1+{\kappa }_{T}{x}^{2})-\frac{1}{2}m{\omega }^{2}{L}^{2}{x}^{2}(1+{\kappa }_{V}{x}^{2})$$

where *m* is the mass of the beam, *L* is its length, *ω* is the resonance frequency of the excited mode, and κ_T_ and κ_V_ are mode-specific constants. The resulting equation of motion is:

13 $$\ddot{x}(1+{\kappa }_{T}^{2})+2{\kappa }_{T}x{\dot{x}}^{2}+{\omega }^{2}(1+2{\kappa }_{V}{a}^{2})x=0$$

If we drive the resonator at a frequency close to its resonance frequency, then $x{\dot{x}}^{2}\sim {\omega }^{2}{x}^{3}\,{\rm{and}}\,{x}^{2}\ddot{x}\sim {\omega }^{2}{x}^{2}\,\,$ and we retrieve equation (1) without the self-oscillation term in the damping function, with *κ* = *κ*_*T*_ + *κ*_*V*_.

Self-oscillation of the resonator is accomplished by placing it in a feedback loop that satisfies Barkhausen’s criterion: the loop gain has a magnitude equal to unity, and the phase shift around the loop is zero or an integer multiple of 2π. There are different ways of fulfilling this criterion, each with different merits regarding the resulting quality of signal (i.e. phase noise) and design complexity. A typical scheme consists of a transimpedance amplifier with gain *R*_*A*_, a phase shifter that introduces a variable phase lag from 0° to 240°, and a buffer that isolates the oscillator circuit from the measuring device.

At resonance, the resonator is characterized by the motional resistance *R*_*M*_. This implies that the gain of the amplifier has to satisfy *R*_*A*_ > *R*_*M*_. In practice, due to additional loss in the phase shifter and parasitic capacitances in the circuit board, *R*_*A*_ should ideally be ~2 *R*_*M*_. Another important factor is the presence of parasitic capacitance in parallel to motional resistance. While nonlinearity is a non-ignorable feature of contour mode resonators, if driving amplitudes are kept small, then its presence can be mitigated. In our simulations, we fixed the value of parameter λ in equation (1) at 0.1, yielding a near-unity dimensionless amplitude of self-oscillation. In practice, *λ* may be tuned by adjusting the gain *R*_*A*_. As our simulations demonstrate the robustness of synchronization in the weakly nonlinear regime, we believe that the nonlinear features of MEMS resonators do not present a significant obstacle towards implementation of the scheme proposed here.

Our noise simulations build in Johnson-Nyquist noise that arises from the self-oscillator electronics at finite temperature *T*; noise sources of a *1*/*f* profile will negligible at the high MHz-range resonance frequencies of MEMS resonators. As our simulations show, the effect of white noise is not detrimental to synchronization for typical perturbing amplitudes that are less than one percent of the self-sustained amplitude of oscillation. This implies a Signal-to-Noise Ratio (SNR) of 100:1 in the output of the feedback electronics will be necessary for proper implementation.

Micromechanical and nanomechanical resonators inherently require low energy for operation. For instance, a four-resonator electrostatically-actuated Fredkin gate was recently demonstrated with an operational energy cost of 10^4^ *k*_*B*_ *T*. This is at least an order of magnitude smaller than the energy required by the standard generation of 22-nm CMOS logic cells^35^. The network of micromechanical oscillators, as discussed here, can be energy efficient by as much as two orders of magnitude by designing both individual resonators and coupling elements for low energy dissipation^14^ and using piezoelectric actuation.

The neurocomputing scheme presented here requires all-to-all coupling between oscillators. While the number of couplings in principle scales as *n* ^*2*^, there exist schemes to reduce this number to just *n* connections^2^. Still, this requirement essentially rules out both direct mechanical and electrostatic coupling via proximity capacitance between resonators. Recent experimental work has achieved synchronization between seven resonator elements via electromagnetic radiation^36^, though external fields cannot easily provide the sort of nonuniform coupling required for pattern storage. Recently, micromechanical oscillators have been wirelessly excited by patch antenna on top of the resonator^37, 38^. This approach enables individual control of each oscillator in the network by suitably designing the corresponding patch antenna. Furthermore, as the storage of information in the oscillator array is accomplished by setting the coupling constants between oscillators such that stored patterns become dynamical fixed points, our simulations have shown that variation of these constants greater than on the order of one part in a thousand can lead to unreliable results. Identifying a scalable, precise, controllable coupling scheme remains as a significant engineering challenge.

Finally, the viability of neurocomputing with MEMS oscillators will be crucially dependent on keeping the spread of natural oscillation frequencies as narrow as possible. Defects in the manufacturing process entail variations in the dimensions of MEMS resonators; current state of the art manufacturing techniques can often only ensure repeatability to within one part in a thousand. Achieving further precision in oscillator frequency will require frequency trimming either by laser or focused-ion-beam after fabrication.

## Conclusion

We have numerically simulated the dynamics of an all-to-all dissipatively coupled system of self-oscillators, storing information in stable synchronized states such that the array is capable of auto-associative memory operation. Feeding in distorted initial conditions, our simulations probe robustness of pattern retrieval in the presence of nonlinearity, frequency and coupling strength dispersion, and white noise perturbations. Our results indicate the need to devise means of ensuring more repeatable resonator frequencies before implementation of the scheme presented here becomes feasible. This can be realized by frequency trimming either by laser or focused ion beam to make the frequency distribution narrow. Tight constraints on the repeatability of coupling strengths will also be required, though we find that synchronization is fairly robust in the presence of stochastic forcing. Overall, our comprehensive numerical studies point to enticing prospects for the hardware realization of MEMS-based neurocomputing elements for memory storage and pattern recognition.

## Electronic supplementary material

Supplementary Information
