# Supplementary material for: Autoassociative Memory and Pattern Recognition in Micromechanical Oscillator Network
Source: Sci Rep. 2017 Mar 24;7:411. doi: 10.1038/s41598-017-00442-y (PMC5428492; doi:10.1038/s41598-017-00442-y)
Supplement: Supplementary file 1 — Supplementary Information [file 41598_2017_442_MOESM1_ESM.pdf]

Supplementary Information: Autoassociative Memory and Pattern Recognition in Micromechanical Oscillator Network

Ankit Kumar[1] and Pritiraj Mohanty[2]

[1]*Department of Physics, California Institute of Technology, 1200 E. California Blvd, Pasadena, CA 91125, USA*
[2]*Department of Physics, Boston University, 590 Commonwealth Avenue, Boston, MA 02215, USA*

1. Derivation of amplitude-phase model

Here, we derive the transformation of the system of ODEs describing the self-oscillator network used in simulation to the amplitude-phase or Hopf normal form generally considered in the literature. The derivation follows[1] (chapter 4). We assume a solution of the form

$$x(t) = \frac{1}{2}(a_i(t)e^{i\omega t} + a_i^*(t)e^{-i\omega t}),\ \frac{\omega - \omega_i}{\omega_i} << 1$$

where a is complex valued, and * denotes complex conjugation. Before substituting into (3), we impose the following condition to ensure we do not increase the number of degrees of freedom into the system:

$$\dot{a}_i(t)e^{i\omega t} + \dot{a}_i^*(t)e^{-i\omega t} = 0$$

then, substituting the first expression into (3)

$$i\omega \dot{a}_i\, e^{i\omega t} - \frac{\omega^2}{2}(a_i e^{i\omega t} + a_i^* e^{-i\omega t}) + \frac{i\omega}{2}(\frac{1}{4}(a_i e^{i\omega t} + a_i^* e^{-i\omega t})^2 - \lambda)(a_i e^{i\omega t} - a_i^* e^{-i\omega t}) +$$
$$\frac{\omega_i^2}{2}(a_i e^{i\omega t} + a_i^* e^{-i\omega t})(1 + \frac{\kappa}{4}(a_i e^{i\omega t} + a_i^* e^{-i\omega t})^2) = \sum_{j=1}^{n} \frac{i\omega p_{ij}}{2}(a_j^{i\omega t} - a_j^* e^{-i\omega t}) + \frac{q_{ij}}{2}(a_j^{i\omega t} + a_j^* e^{-i\omega t})$$

Expanding and simplifying,

$$i\omega \dot{a}_i - (\frac{\omega^2 - \omega_i^2}{2})(a_i + a_i^* e^{-2i\omega t}) - \frac{i\omega\lambda}{2}(a_i - a_i^* e^{-2i\omega t}) + \frac{i\omega}{8}(a_i^3 e^{2i\omega t} + a_i |a_i|^2 - a_i^* |a_i|^2 e^{-2i\omega t} - a_i^{*3} e^{-4i\omega t})$$
$$+ \frac{\kappa\omega_i^2}{8}(a_i^3 e^{2i\omega t} + 3a_i |a_i|^2 + 3a_i^* |a_i|^2 e^{-2i\omega t} - a_i^{*3} e^{-4i\omega t}) = \sum_{j=1}^{n} \frac{i\omega p_{ij}}{2}(a_j - a_j^* e^{-2i\omega t}) + \frac{q_{ij}}{2}(a_j + a_j^* e^{-2i\omega t})$$

Now, assuming that the amplitude is modulated on a timescale much slower than that given by the oscillations at frequency ω, we can average the equation over the period corresponding to ω, over which terms proportional to $e^{\pm 2i\omega t}$ and $e^{\pm 4i\omega t}$ average to zero. This leaves the following:

$$\dot{a}_i = (\frac{\lambda}{2} - i\frac{\omega^2 - \omega_i^2}{2})a_i - (\frac{1}{8} - \frac{3i\kappa\omega_i^2}{8\omega})a_i |a_i|^2 + \sum_{j=1}^{n}(\frac{p_{ij}}{2} + \frac{q_{ij}}{2i\omega})a_j$$

which is equation (4).

2. Complete set of patterns stored in oscillator network

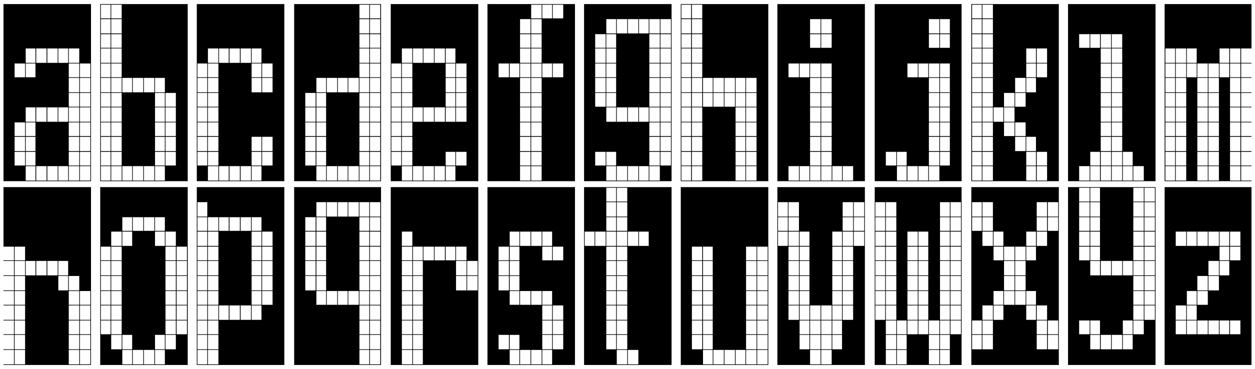

Figure S1: *2-D Visualization of the vector set* $\{\xi^{\mu}\}$ *where each* $\xi^{\mu}{}_{i}$ *= +/- 1 has been set to white and black, respectively stored in our oscillator array during simulation. Storage is achieved by setting the coupling matrix of the oscillator array according to equation (9). The initial conditions of simulation are obtained by starting from a stored pattern and randomly flipping pixels until the desired initial degree of match is obtianed.*

3. Effective stiffening of system response with iterated nonlinearity

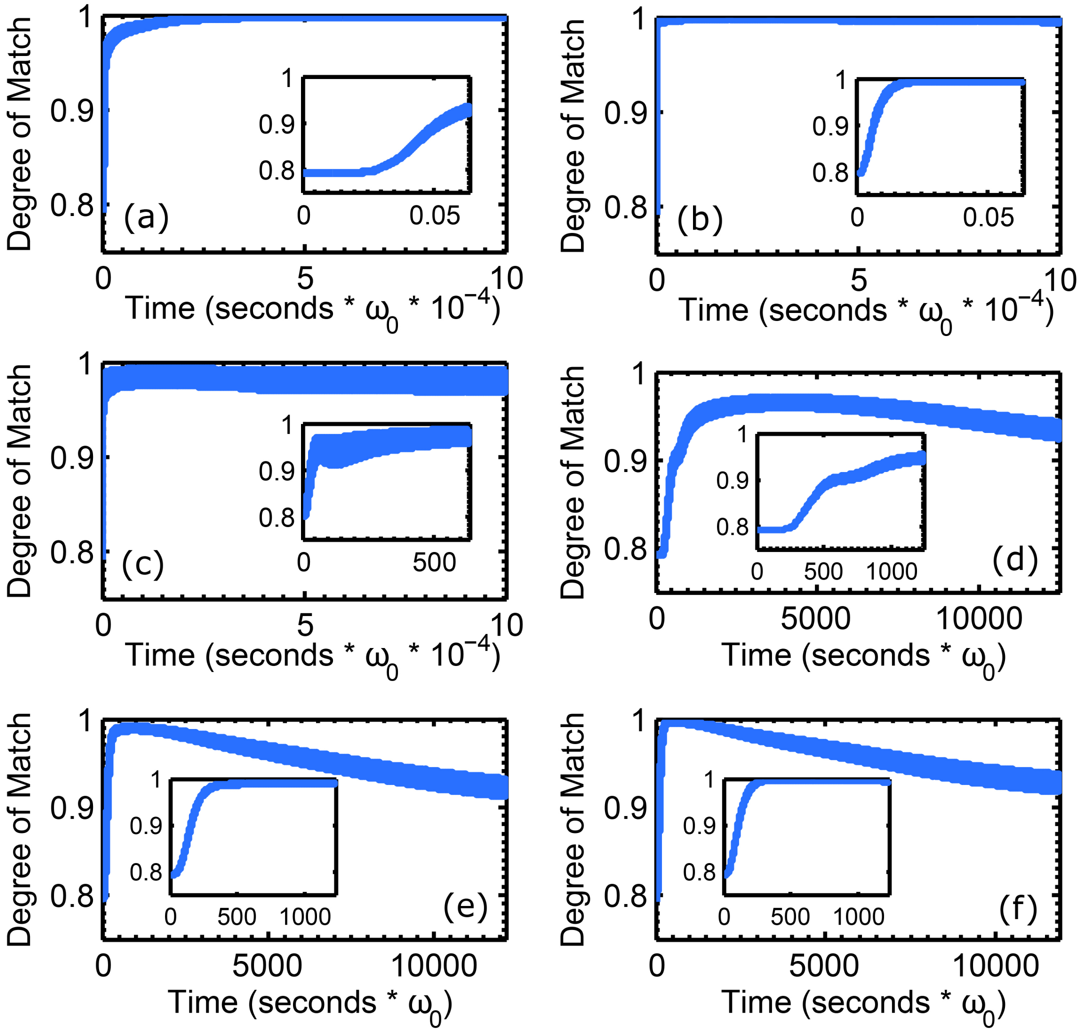

Figure S2: *Degree of match with the `a' pattern. Insets show the first 500-1000 normalized seconds. (a – c) Long timescale simulations in the absence of frequency dispersion but incorporating the nonlinear term demonstrate stability of synchronization ((a): κ = 0, (b): κ = 0.5, (c): κ = 2). Increasing the nonlinearity parameter quickens the response of the system. Large values of nonlinearity give oscillations in the degree of match parameter, but these oscillations remain bounded over time. (d – f) Frequency distributions with a width of* $\delta = 5 * 10^{-4}$ *are introduced. Nonlinearity again stiffens the response of the system ((a): κ = 0, (b): κ = 0.1, (c): κ = 0.2). Notably, however, the loss of synchronization over time when oscillator frequencies are incommensurate is present both with and without nonlinearity.*

4. Response of individual self-oscillator to varying degrees of stochastic forcing

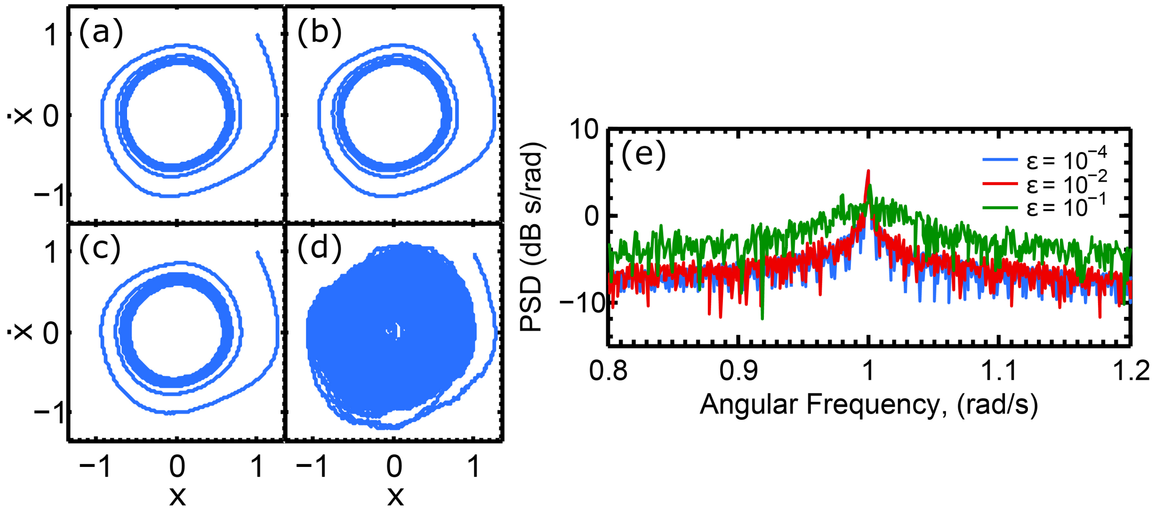

Figure S3: *(a)-(d) Phase space plots (displacement versus velocity) of a single unity amplitude self-sustaining oscillator for 4 different levels of stochastic forcing. Starting top left and moving clockwise:* $\varepsilon = 10^{-4}$, $\varepsilon = 10^{-3}$, $\varepsilon = 0.1$, $\varepsilon = 0.01$. *Periodicity is unsustainable at* $\varepsilon = 0.1$. *(e) Corresponding power spectral density for a single oscillator subject to noise. The responses for* $\varepsilon = 0.01$ *and* $\varepsilon = 10^{-4}$ *demonstrate roughly the same profile, though additional sideband noise is present in the former case. Again, at* $\varepsilon = 0.1$*, the average noise amplitude is only one order of magnitude less than the oscillation amplitude, and periodicity is highly surpressed. Importantly, at realistic noise amplitude, there does not seem to be significant distrubrance of periodic behavior.*

5. Reproduction of results with random pattern storage

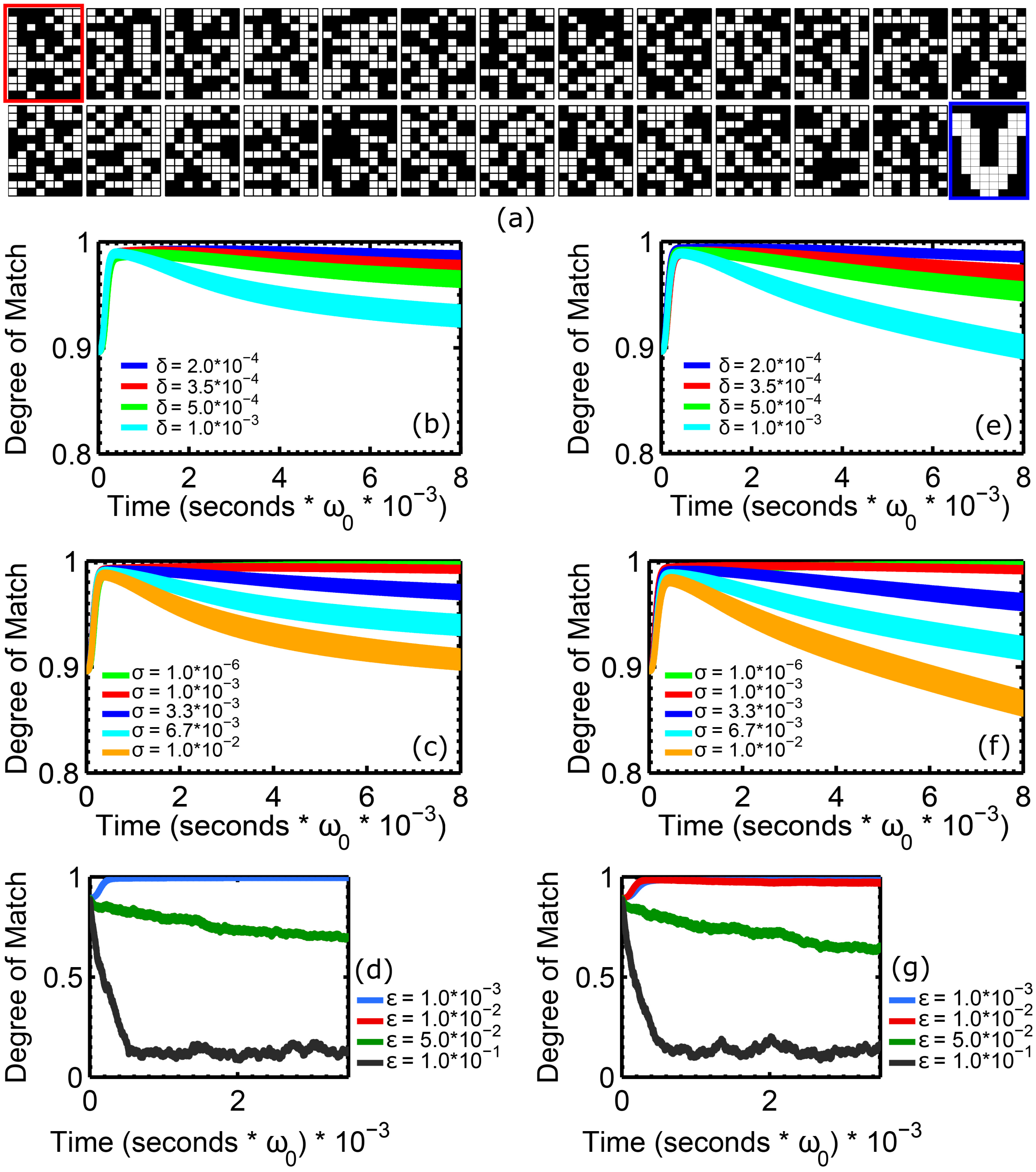

Figure S5: *In order to demonstrate a lack of sensitivity of the main results to the stored pattern set and final target pattern, we duplicate them for both a randomly generated stored pattern set, and for the "v" letter of the alphabet set of Figure S1. (a) The first row and first 12 patterns are 25 randomly generated patterns stored in the oscillator matrix, with a distorted version of the pattern highlighted in red fed in as an initial condition when generating data for figures (b), (c), and (d). Distorted versions of the "v" pattern highlighted in blue are set as initial conditions with the alphabet set of Figure S1 stored when generating data for figures (e), (f), (g). Figures (b) and (e) are reproductions of Figure 3(a), (c) and (f) of Figure 3(b), and (d) and (g) of Figure*

4. There are no significant deviations in the simulation results from those presented in Figures 3 and 4. Figures (b) and (e) show that the degree of match parameter does not remain stable for large frequency distribution widths. Similarly, figures (c) and (f) show a similar effect for distribution of the coupling strengths, again exhibiting a tolerance of an order of magnitude larger width in this distribution as compared to the frequency distribution. Figures (d) and (g) exhibit tolerance to perturbations by noise with amplitude up to $\varepsilon = 1.0 * 10^{-2}$.

---

[1] A. Balanov, N. Janson, D. Postnov, and O. Sosnovtseva, Synchronization: From Simple to Complex, Springer Series in Synergetics (Springer, 2009).
